# Delineation of the phenotypes and genotypes of facial infiltrating lipomatosis associated with PIK3CA mutations

**DOI:** 10.1186/s13023-023-02786-3

**Published:** 2023-07-14

**Authors:** Hongrui Chen, Bin Sun, Wei Gao, Yajing Qiu, Chen Hua, Xiaoxi Lin

**Affiliations:** Department of Plastic and Reconstructive Surgery, Shanghai Ninth People’s Hospital, Shanghai Jiao Tong University School of Medicine, 639 Zhizaoju Road, Shanghai, 200011 People’s Republic of China

**Keywords:** Facial infiltrating lipomatosis, PIK3CA mutations, Phenotypes, Genotypes

## Abstract

**Background:**

Facial infiltrating lipomatosis (FIL) is a rare congenital disorder characterized by unilateral facial swelling, for which surgery is the prevailing therapeutic option. Several studies have shown that the development of FIL is closely associated with PIK3CA mutations. This study aimed to further identify rare clinical features and underlying molecular variants in patients with FIL.

**Results:**

Eighteen patients were included in this study, and all patients presented with infiltrating adipose tissues confirmed by magnetic resonance imaging. Macrodactyly, polydactyly, hemimegalencephaly and hemihyperplasia were also observed in patients with FIL. In total, eight different PIK3CA mutations were detected in tissues obtained from sixteen patients, including the missense mutations p.His1047Arg (n = 4), p.Cys420Arg (n = 2), p.Glu453Lys (n = 2), p.Glu542Lys (n = 2), p.Glu418Lys (n = 1), p.Glu545Lys (n = 1), and p.His1047Tyr (n = 1) and the deletion mutation p.Glu110del (n = 3). Furthermore, the GNAQ mutation p.Arg183Gln was detected in the epidermal nevus tissue of one patient. Imaging revealed that several patients carrying hotspot mutations had more severe adipose infiltration and skeletal deformities.

**Conclusions:**

The abundant clinical presentations and genetic profiles of FIL make it difficult to treat. PIK3CA mutations drive the pathogenesis of FIL, and PIK3CA hotspot mutations may lead to more extensive infiltration of lipomatosis. Understanding the molecular variant profile of FIL will facilitate the application of novel PI3K-targeted inhibitors.

## Introduction

Facial infiltrating lipomatosis (FIL) is a congenital facial deformity characterized by unilateral facial enlargement that progressively worsens with age. Capillary blush, mucosal neuroma, macrodontia and macroglossia are also frequently observed in patients with FIL [1]. Histologically, hyperproliferative adipose tissues without a tendency for malignant transformation can infiltrate the surrounding muscles and parotid glands, sometimes accompanied by an increase in the number of blood vessels [2]. Moreover, patients may suffer from aesthetic abnormalities that seriously affect their daily life and psychological happiness. A combination of liposuction, debulking, facial nerve anatomy and osteotomy is the main approach to treat FIL, but the risk of damage to the facial nerves and postoperative recurrence limit the effectiveness of surgery [2, 3].

The aetiology of FIL remained unclear until somatic phosphatidylinositol 3-kinase catalytic subunit alpha (PIK3CA) gain-of-function mutations were detected in multiple tissues from FIL patients [4, 5]. PIK3CA encodes the p110α catalytic subunit of IA phosphatidylinositol 3-kinase (PI3K), and gain-of-function PIK3CA variants enhance the activity of PI3K and further promote the phosphorylation of downstream molecules, including protein kinase B (AKT) and mammalian target of rapamycin (mTOR), resulting in overactivation of the PI3K-AKT-mTOR pathway, which is directly associated with cell growth, survival and proliferation [6]. PIK3CA variants have been identified in a range of segmental overgrowth disorders or severe syndromes, and these diseases, including FIL, have been summarized by the acronym “PROS” (PIK3CA-related overgrowth spectrum) [7]. Among the numerous PIK3CA mutations that have been detected in PROS, p.Glu542Lys, p.Glu545Lys and p.His1047Arg are considered hotspot mutations, as they are highly recurrent and account for more than 80% of PROS cases [8].

Although many articles have described the clinical features of FIL, the genotypic profile of FIL has not yet been established, and the effects of different base and amino acid alterations on the severity of FIL are not fully understood. Here, we collected clinical information from 18 patients with FIL who presented to our department and underwent genetic testing. Our data further confirm that PIK3CA mutation is a crucial factor driving the occurrence of FIL, and specific variants may be associated with more severe conditions.

## Results

### Clinical presentation

A total of eighteen patients were included in this study, and their clinical data are summarized in Table 1. The median age of the patients when they first came to our department was 14.5 years (ranging from 1 to 43), and the group included eleven males and seven females. All patients were observed to have facial asymmetry with variable severity at birth, and the asymmetry became more pronounced with age. Twelve patients had lesions on the right side, and six had lesions on the left side. All patients were new cases with no family history of facial enlargement or limb overgrowth.

**Table 1:** Common clinical characteristics of FIL

No	Sex	Age	Side	EN	MD	MG	TL	OOBP
1	M	2	R	−	−	−	Lower lip	Right hemimegalencephaly, enlargement of right lower limb and foot
2	F	16	L	+	−	+	Both	–
3	M	5	R	+	−	−	Lower lip	–
4	M	20	R	−	−	−	Lower lip	–
5	M	14	R	+	+	+	Both	Right megalencephaly
6	F	18	L	+	−	+	–	–
7	F	27	R	−	−	+	Lower lip	–
8	M	23	R	+	+	+	Lower lip	–
9	M	3	L	+	−	−	Lower lip	–
10	F	4	L	−	−	−	–	–
11	F	17	R	−	+	+	Lower lip	–
12	F	43	R	−	−	−	–	–
13	M	13	R	−	−	−	Lower lip	–
14	M	2	R	−	+	+	Lower lip	–
15	M	1	L	+	+	+	Lower lip	Macrodactyly, polydactyly
16	M	2	R	+	−	+	Lower lip	Right megalencephaly and bilateral cortical thickening of the cerebellar hemispheres
17	F	15	R	+	−	−	Lower lip	–
18	M	24	R	−	−	+	–	Macrodactyly, lipoma on abdomen, capillary malformation in abdomen and lower limb

*M* Male, *F* Female, *L* Left, *R* Right, *EN* Epidermal nevi, *MD* Macrodontia, *MG* Macroglossia, *TL* Thick lip, *OOBP* Overgrowth of other body parts

**Fig. 1:**
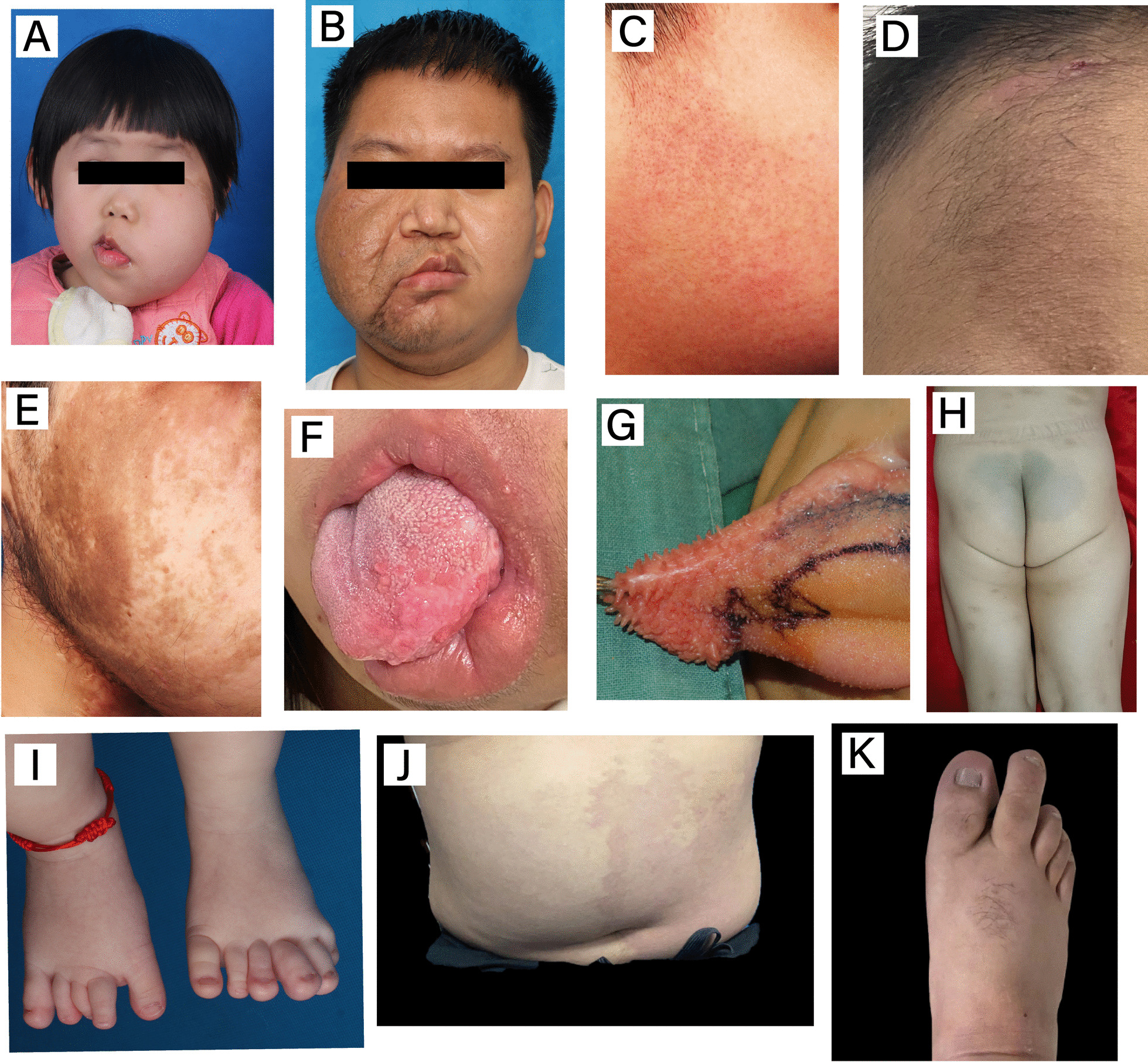
Common clinical presentations of FIL. **A**, **B** The appearance of FIL, with facial asymmetry and unilateral facial swelling, is the most visual first impression. As shown in (**A**), the patient’s skin may be tighter due to the growth of the mass. The skin may also be looser overall, as shown in (**B**). The thickened lip is usually located on the affected side and can involve both the upper and lower lips, but in this study, there was a higher percentage of lower lip involvement. **C** Capillary blush in front of the ear. **D**, **E** Epidermal nevi can occur in several areas on the affected side, including the forehead, cheeks, and in front of the ears. Sometimes, these areas are also accompanied by excessive hair growth. **F**, **G** Hemimacroglossia and lip thickening are crucial elements in the pathology examination. Tongue thickening can manifest as thickening of the tongue body and enlargement of the tongue papillae. **H** The right thigh of Patient #1 was thicker than the contralateral side. **I** Patient #15 had polydactyly and macrodactyly, which is extremely rare in patients with FIL. **J**, **K** Patient #18 had a lipoma and capillary malformation in the abdomen, and he also had macrodactyly on the affected side

All the clinical features recorded are shown in Fig. 1. Skin changes were usually the first symptoms to be observed. Cutaneous capillary malformations were not a frequent finding (3/18), which was inconsistent with previous reports in the literature [1]. However, epidermal nevi, another type of lesion often observed alone or in other diseases within PROS [9], were seen in many patients with a variable distribution (9/18), which was seldom described in previous articles. Three patients showed an increased number of facial hairs, most of which were distributed in areas with pigmentation.

Notably, although patients with FIL have a core presentation of fatty hyperplasia and infiltration, intraoral abnormalities such as abnormal tooth development and tongue enlargement also occur [1, 10]. We used photographs, digital dental radiographs and computed tomography (CT) three-dimensional (3D) reconstructions to determine if the patient had tooth problems. Five (28%) patients had irregular tooth arrangements, and five (28%) patients had macrodontia. Macroglossia, especially thickening of the affected hemiglossia, was observed in 50% of cases (9/18), and enlargement of the lingual papillae was also observed together with thickening of the tongue body (1/18). Twelve (66%) patients had thickened lips; two patients had thickened upper and lower lips, and ten patients had thickened lower lips only. Lip thickening was usually located on the affected side and did not cross the midline of the lip or involve the entire lip.

The patients with FIL had combinations of other individualized features, and this was likely caused by the sporadic distribution of somatic mosaic PIK3CA mutations. For example, Patient #1 was found to have an enlarged right leg and foot in addition to an enlarged right face and was considered to possibly meet the criteria for hemihyperplasia. Patient #11 was found to have hearing loss on the affected side. Macrodactyly, a disease also associated with PIK3CA mutations, was observed in two patients. Patient #15 also had polydactyly in his left foot and macrodactyly in both feet, and Patient #18 had cutaneous capillary malformations, macrodactyly, and a lipoma in the abdomen. We considered whether Patient #18 was a case of CLOVES syndrome (congenital lipoma overgrowth, vascular malformation, epidermal nevi, scoliosis), but he did not have scoliosis and therefore did not meet the diagnostic criteria.

### Imaging findings

**Fig. 2:**
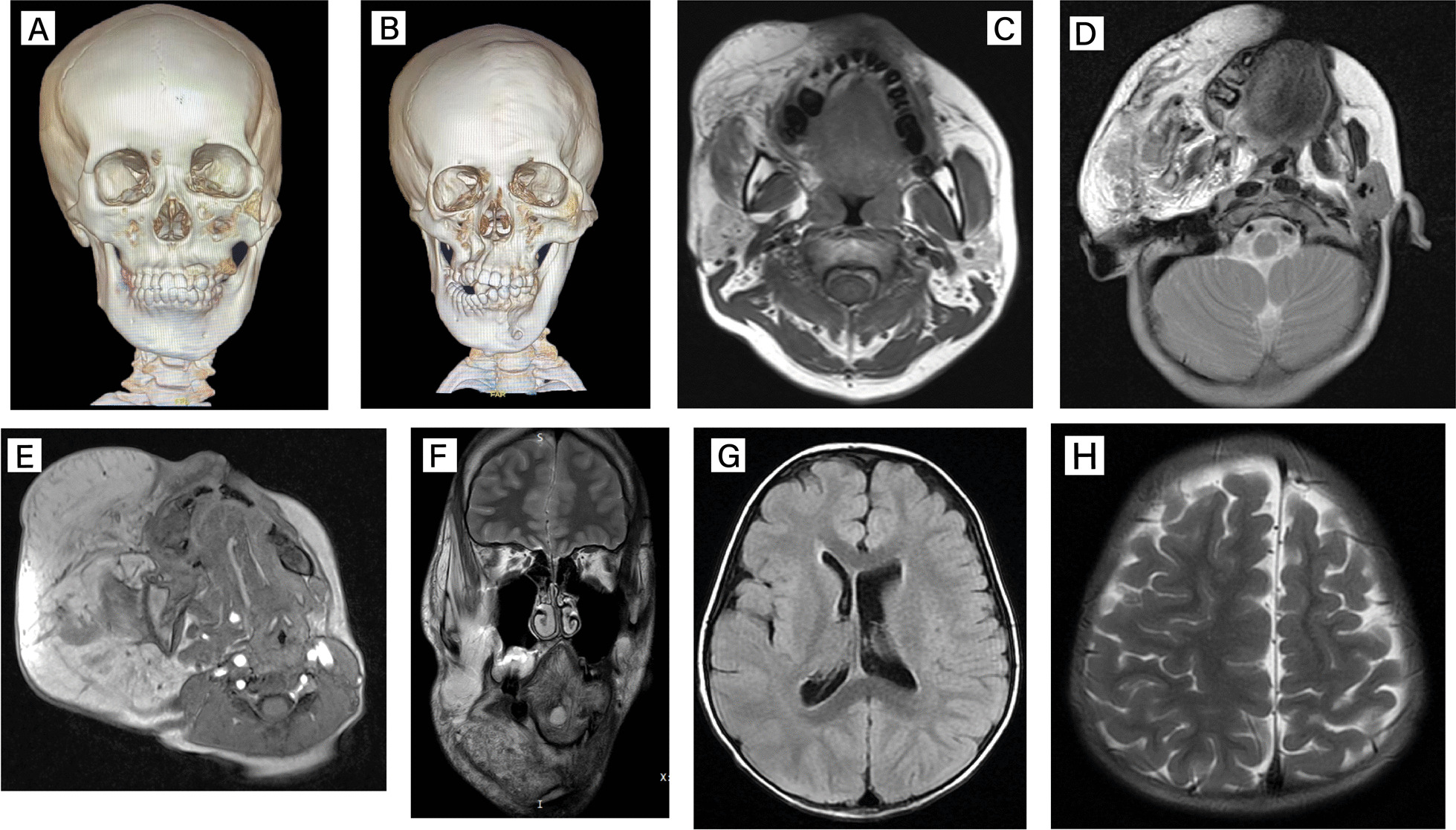
Maxillofacial imaging findings. **A** Stereoscopic image of the skeleton of Patient #6 provided by CT 3D reconstruction. Most patients had potential skeletal overgrowth, including zygomatic, maxillary, and mandibular bones. **B** The 3D reconstructed image of Patient #11. Excessively large lipomatosis can compress the surrounding structures, causing the bones to develop malformations while overgrowing and interfering with the normal growth and alignment of the teeth. **C**–**E** Magnetic resonance (MR) images of Patients #8, #7, and #11. Infiltrative growth of lipomatosis can have a variable impact. As shown in C, enlarged occlusal muscles and parotid glands are very prominent features, and mild infiltration of adipose tissue does not affect the normal anatomy of the maxillofacial region. As shown in (**D**, **E**), more severe diffuse infiltration of adipose tissue makes facial structures confusing and difficult to distinguish and can be detrimental to the patient’s function. **F** T2 coronal image of Patient #5. The aberrant signal and structure near his right mandible seemed to indicate that this was a lymphatic malformation. **G**, **H** Cranial MRI of Patient #1 revealed asymmetric brain development and an enlarged left ventricle

The radiographic features of the patients are summarized in Table 2. The maxillofacial skeleton was often affected [11], and skeletal deformities were classified into two categories, one with hyperplasia of the skeleton and the other with distortions caused by compression. CT 3D reconstructions demonstrated that the zygomatic bone was the most likely to be affected of all maxillofacial bones, with ten (56%) patients exhibiting zygomatic hyperostosis. Six (33%) patients had maxillary augmentation, and in two other patients, the maxilla appeared to be depressed and displaced contralaterally by soft tissue compression, resulting in a deviation of the upper lateral dentition. Mandibular overgrowth was also observed in four (22%) patients, and Patient #11 also had an extruded deformity of the mandible (Fig. 2).

**Table 2:** Maxillofacial imaging findings

No	Zygoma	Maxilla	Mandible	Masseter	Medial pterygoid	Lateral pterygoid	Parotid gland
1	N	N	N	E	N	N	E, I
2	E	N	N	E, I	N	E	E, I
3	E	N	N	I	N	N	N
4	E	N	N	E, I	N	N	E, I
5	E	E, D	E	E, I	E, I	E, I	E, I
6	E	D	E	E, I	E	E	E, I
7	D	E, D	N	E, I	E, I	E, I	E, I
8	N	N	N	I	N	E	E
9	N	N	N	E	N	N	E
10	N	N	N	I	N	N	I
11	E	D	D	E, I	E, I	I	E, I
12	N	N	N	E	N	N	E
13	E	N	N	E, I	N	N	E, I
14	E	D	E	E, I	E, I	E, I	E, I
15	E	E	E	E, I	N	I	E, I
16	N	N	E	N	N	N	E
17	E	N	E	E, I	E	N	I
18	E	N	E	N	N	N	E, I

The deformity is only used to describe the maxillofacial skeleton, which means that the skeleton has lost its normal morphology due to soft tissue compression. Deformity should be distinguished from enlargement, which is only used to describe bones with localized hyperplasia

*N* Normal, *E* Enlargement, *D* Deformity, *I* Infiltrated

Magnetic resonance imaging (MRI) identified that the occlusal muscles in all patients were affected and characterized by enlargement alone (5/18), infiltration alone (3/18), or both enlargement and infiltration (10/18). In addition to the masticatory muscles on the outside of the mandible, the internal pterygoid muscle (5/18) and the external pterygoid muscle (8/18), located on the medial side of the mandible, were also invaded by adipose tissues, but infiltration of the temporalis muscle was observed only in Patient #6. Parotid infiltration was very prominent, with signals of adipose tissues visible in thirteen (72%) patients, suggesting that they all contained an infiltrating fatty component. Inferior turbinate hypertrophy was observed in five (27%) patients.

MRI of three (17%) patients also revealed hemimegalencephaly (HMEG), coincidentally located on the right side in all three patients. Patient #1 had asymmetrical brain development with an enlarged left ventricle, and abnormal signals were also seen in the right occipital lobe, which was thought to be cortical dysplasia (Fig. 2). Enlargement of the right cerebral hemisphere and disorganization of the sulcal gyrus with unsmooth grey and white matter demarcation were observed in both Patient #5 and Patient #16.

### Genotypic characteristics

**Fig. 3:**
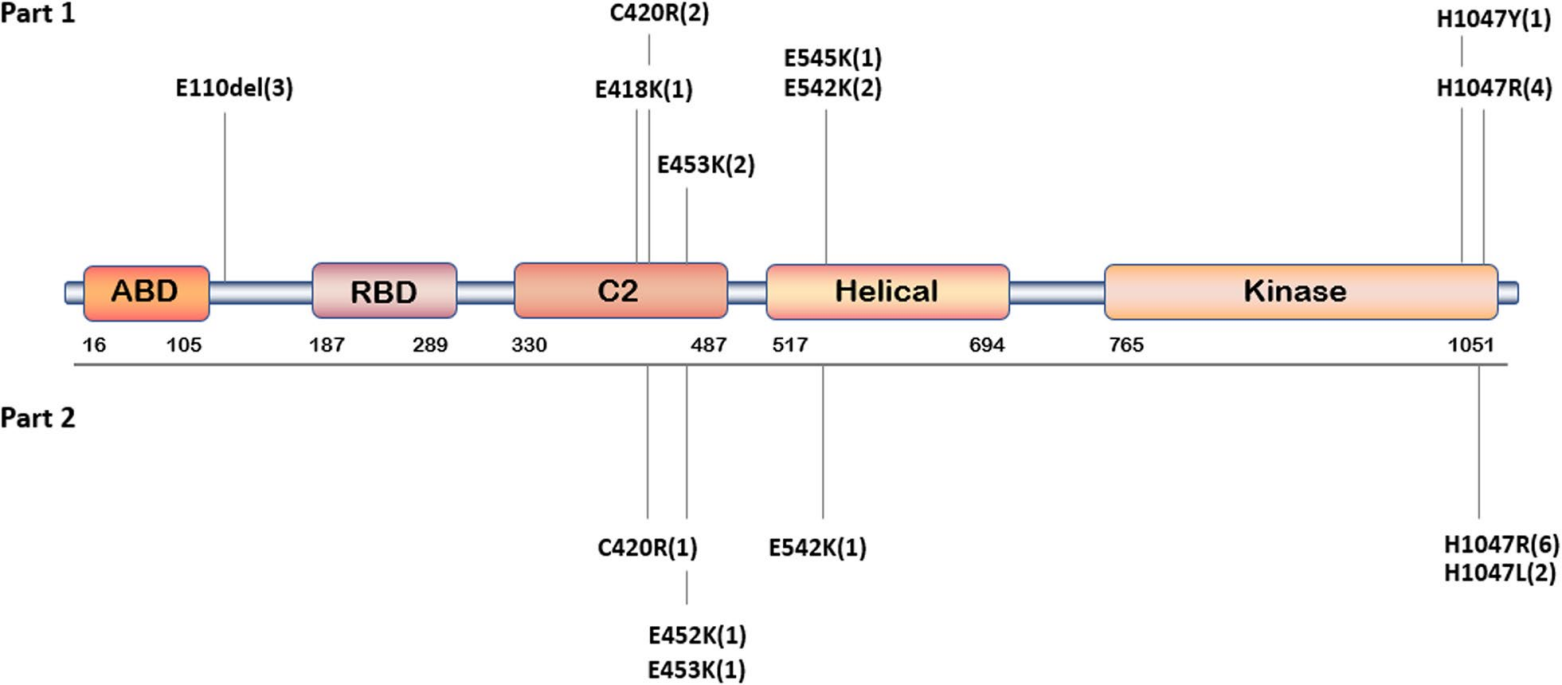
Overview of the distribution of PIK3CA mutant loci. The distribution of the PIK3CA variants identified in this study and the previously reported mutant loci. Part 1: A total of eight mutations were identified in 18 patients, distributed in the C2, helical, and kinase domains of p110α. Part 2: The variants associated with FIL that have been reported thus far. We found four additional PIK3CA mutations related to FIL in patients from our centre

The genetic information of all patients is shown in Table 3, and the distribution of the different mutation sites in the p110α protein is shown in Fig. 3. The following types of tissue samples were used for molecular confirmation: epidermal nevus (n = 3), skin and subcutaneous fat obtained by biopsy needle (n = 6), and surgically obtained fat (n = 9). Previous studies reported that the detection rate of mutations in blood samples was extremely low in non-central nervous system (CNS) disorders [12], so we only used tissue samples for analysis. A total of eight PIK3CA mutations were detected in tissues isolated from sixteen (89%) patients with variant allele fractions (VAFs) ranging from 2.35% to 26.50%, and the average VAF was 12.76%. Patient #14 was molecularly confirmed elsewhere, and his report did not provide an accurate VAF value. The most frequent mutation detected was p.His1047Arg (n = 4), followed by p.Glu110del (n = 3), p.Cys420Arg (n = 2), p.Glu453Lys (n = 2) and p.Glu542Lys (n = 2). Three other mutations, p.Glu418Lys, p.Glu545Lys and p.His1047Tyr, were detected in one patient. All detected mutations were missense mutations in PIK3CA except for p.Glu110del, which was a deletion mutation and was found in three patients. Patient #10 had a negative result, and a GNAQ mutation was detected in the epidermal nevus obtained from Patient #16.

**Table 3:** Detailed genetic information

No	Variant gene	Sample	Exon	Base alteration	Amino acid change	Variant allele fraction (%)
1	PIK3CA	ATFS	exon8	c.1252G > A	p. Glu418Lys	19.80
2	PIK3CA	SSF	exon10	c.1624G > A	p. Glu542Lys	11.29
3	PIK3CA	SSF	exon8	c.1258 T > C	p. Cys420Arg	2.35
4	PIK3CA	ATFS	exon21	c.3140A > G	p. His1047Arg	7.99
5	PIK3CA	EN	exon8	c.1258 T > C	p. Cys420Arg	22.69
6	PIK3CA	ATFS	exon10	c.1624G > A	p. Glu542Lys	6.48
7	PIK3CA	ATFS	exon21	c.3140A > G	p. His1047Arg	12.20
8	PIK3CA	SSF	exon8	c.1357G > A	p. Glu453Lys	24.24
9	PIK3CA	ATFS	exon2	c.328_330delGAA	p. Glu110del	26.50
10	–	SSF	–	–	–	–
11	PIK3CA	SSF	exon21	c.3140A > G	p. His1047Arg	6.37
12	PIK3CA	SSF	exon8	c.1357G > A	p. Glu453Lys	12.26
13	PIK3CA	ATFS	exon2	c.328_330delGAA	p. Glu110del	11.89
14	PIK3CA	ATFS	exon21	c.3140A > G	p. His1047Arg	–
15	PIK3CA	ATFS	exon2	c.328_330delGAA	p. Glu110del	14.11
16	GNAQ	EN	exon4	c.548G > A	p. Arg183Gln	7.05
17	PIK3CA	EN	exon10	c.1633 > A	p. Glu545Lys	10.53
18	PIK3CA	ATFS	exon21	c.3139C > T	p. His1047Tyr	2.63

*ATFS* Adipose tissues obtained from surgery, *EN* Epidermal nevi, *SSF* Skin and subcutaneous fat

All variants found in this study have been previously reported in the National Center for Biotechnology Information (NCBI) database (https://www.ncbi.nlm.nih.gov/clinvar/) in PROS, but four mutations were identified for the first time in FIL (p.Glu110del, p.Glu418Lys, p.Glu545Lys, and p.His1047Tyr). The catalytic subunit p110α of the PI3K protein has a total of five functional domains: the adaptor-binding domain (ABD), Ras-binding domain (RBD), C2 domain, helical domain, and kinase domain. Among them, the C2 domain, helical domain and kinase domain are the major regions of the inhibitory regulatory subunit p85 of the PI3K protein, and mutations in these regions have a substantial impact on the function of PI3K [6]. All the variants found in this study occurred in these three structural domains except for one deletion mutation, which was located between the ABD and RBD. The PIK3CA mutations p.Glu418Lys, p.Cys420Arg, and p.Glu453Lys were in the C2 domain, p.Glu542Lys and p.Glu545Lys occurred in the helical domain, and p.His1047Arg was present in the kinase domain. Only 39% (7/18) of the patients harboured a PIK3CA hotspot mutation.

### Association between phenotypic features and genotypic characteristics

Analysing the correlation between genotypes and phenotypes helps to broaden our knowledge of FIL. In the published literature focusing on FIL, there was no clear definition to describe the severity of FIL. Since it was difficult to accurately evaluate the severity of phenotypic variants in patients, we decided to classify the overall severity by comparing the imaging findings of patients carrying PIK3CA mutations. We have described the imaging findings of several anatomic structures in Table 2. According to the imaging findings, we first tried to identify the correlation between the types of mutations and the number of affected structures.

Three hotspot mutations, p.Glu542Lys, p.Glu545Lys, and p.His1047Arg, were classified as gain-of-function mutations with strong oncogenicity based on the tumorigenic ability of the mutations, and the rest were classified as moderate or weakly oncogenic [13], so we first analysed the differences in the effects of the hotspot/nonhotspot mutations on tissue infiltration and proliferation. The average number of affected tissues was 5.8 for patients carrying hotspot mutations compared to 3.4 for other patients, which was a significant difference (*P* = 0.032), suggesting that patients with FIL carrying hotspot mutations tended to have more maxillofacial anatomical structures affected by lipomatosis and presented a higher overall severity. However, we observed that the different molecular variants were not significantly associated with the generation of the clinical phenotypes recorded in Table 1. In addition, we analysed the VAF and the number of affected structures, but there was no significant association between them.

## Discussion

FIL deserves attention because of the complexity of its aetiology, the serious damage it causes to facial appearance and function, and the limitations of the curative approach. Diffuse lesions on one cheek will invade the surrounding tissues with age, leading to muscle and parotid gland involvement. Problems in the oral cavity, such as macrodontia, poor root formation, missing permanent teeth, malocclusion of large teeth, and premature eruption of teeth, are also common concerns in patients with FIL [14]. Underlying localized skeletal hyperplasia and sclerosis, which may involve the skull, zygomatic arch, mandible, and cervical spine [15] and, more rarely, temporomandibular joint ankylosis may occur [16]. The overlap of all of the above factors makes it easy to misdiagnose the patient with hemifacial hyperplasia (HFH). HFH is another congenital deformity that causes unilateral facial enlargement and involves multiple tissues. It is typically characterized by hyperplasia rather than hypertrophy because the increase in cell number is more significant than the increase in cellular volume [17]. HFH is further divided into true HFH, in which all tissues are enlarged, and partial HFH, in which not all structures are involved or enlarged to the same degree [18]. Both FIL and HFH have no family history and are not associated with delivery patterns. They can be observed at birth or several weeks later and progress rapidly during adolescence. It is sometimes difficult to clearly distinguish between the two diseases, and the presence or absence of diffuse infiltrative growth of adipose tissue is an important basis for the differential diagnosis. However, some scholars believe that FIL is a subtype of HFH [10]. Our study focused on FIL, and infiltration was observed on imaging in all included patients.

The combination of FIL with concurrent HMEG on the affected side has been reported in several cases. Patients may present with intractable epilepsy that is difficult to control with medication, and cranial MRI may reveal that the patient has pachygyria and agenesis of the corpus callosum [19]. We recommend that MRI examination of outpatients who are suspected of FIL should also include a scan of the brain. The presence of fatty masses together with HMEG is also seen in epidermal nevus syndrome (ENS) and encephalocraniocutaneous lipomatosis (ECCL), and some articles have suggested that FIL with HEMG is a specific subtype of these syndromes [20]. Identifying features of ENS include overgrowth of soft and hard tissue in the head and face, vascular malformations, and cerebral malformations [21]. In contrast, ECCL is associated with unilateral cerebral, cutaneous, and subcutaneous tissue and ocular anomalies, and the lipomas seen in ECCL not only are located subcutaneously but can also occur intracranially and in the spinal canal, without infiltrative properties [22]. We can speculate that the coexistence of FIL and HMEG may be because the first mutant cell arises early in ectodermal formation since both the CNS and the epidermis are derived from the ectoderm. However, if the mutation occurs after neural tube formation, hyperproliferation usually occurs at only one site, i.e., FIL alone or HMEG alone. It has also been suggested that aberrant neural crest cell formation, migration, or differentiation are common causes of these neurocutaneous syndromes and that dysregulation of pluripotent neural crest cells may lead to terminal overgrowth of adipose tissue [23].

Considering the crucial role of the PI3K-AKT-mTOR signalling pathway in regulating cell proliferation, metabolism, apoptosis, and the cell cycle [6], activating mutations of the proteins in this signalling pathway can have a significant impact on normal cellular life activities, producing malignant tumours as well as other benign overgrowth syndromes [24], such as PROS or Proteus syndrome (PS) associated with AKT1 mutations. PROS is a broad-spectrum concept proposed in 2014 that integrates multiple sporadic or mosaic overgrowth disorders associated with PIK3CA mutations, including CLOVES syndrome, Klippel-Trenaunay syndrome (KTS), macrodactyly, megalencephaly capillary malformation-polymicrogyria (MCAP), HMEG, muscle hemihyperplasia (MHH), and hemihyperplasia multiple lipomatosis (HHML) [25]. FIL is also included in PROS because of the occurrence of PIK3CA mutations [4]. Mesodermal-derived tissues such as bone, fat, skin, muscle, and blood vessels are the most commonly affected tissues among PROS patients [26], and other overlapping features include the mostly congenital and disproportionately asymmetric nature of these syndromes [24]. PS is another type of localized overgrowth caused by AKT1 gain-of-function mutations involving both limbs and soft tissues (e.g., fat and blood vessels), with a similar evolutionary process to PROS, and the identification of PS is particularly dependent on the results of genetic testing [27]. PS also involves maxillofacial changes, such as a long face and dolichocephaly [28], and some early case reports also described PS with fatty masses in the maxillofacial region, which now is more indicative of FIL [29]. The identification of PS and FIL relies on molecular evidence. Except for one negative case and one GNAQ mutation, all other patients diagnosed with FIL in our study were found to have gain-of-function PIK3CA mutations, which further confirms the relevance of PIK3CA variants to FIL and affirms that FIL should be distinguished from other diseases causing facial enlargement. We were surprised by the detection of GNAQ mutations in epidermal nevus tissue from Patient #16 since these mutations are mainly seen in Struge-Weber syndrome, port-wine stains, and uveal melanoma [30, 31], and few articles have reported that GNAQ mutations can cause epidermal nevi. One possible explanation is that GNAQ mutations affect the MEK pathway, which is associated with epidermal nevi [32]. It is unclear whether GNAQ mutations are related to the development of FIL, and this requires further discussion and validation. HMEG has also been associated with PIK3CA mutations [33], but we did not perform biopsies of brain tissue in patients with HMEG, considering the risks of surgery.

A prominent feature of PROS is its rich clinical phenotypes and genotypes; that is, the same PIK3CA variants can generate distinct phenotypic presentations, and similar clinical manifestations can be caused by multiple variants [34]. The impact of variants on downstream signalling, VAF, and specific tissue involvement are all factors that influence the clinical phenotypes, but the detailed mechanisms have not been fully elucidated. Recent studies have shown that nonhotspot mutations occur more frequently in CLOVES syndrome [35], and some less common mutant loci have been observed only in MCAP [36, 37], while some mutations possessing strong oncogenic capacity were detected in somatic segmental overgrowth disorders [27]. Another recent study that included many cases yielded the same conclusion [12]. CLOVES is a systemic syndrome that includes a variety of manifestations: congenital lipomatous overgrowth (CLO), vascular malformation (V), epidermal nevi (E), and scoliosis/spinal malformation (S) [9]. MCAP, on the other hand, is characterized by megalencephaly, capillary malformations, and overgrowth in other body parts as the primary presentation [37]. Both diseases are more severe and widespread forms of PROS, and both are closely associated with nonhotspot mutations. A possible explanation for this is that nonhotspots, due to their weaker pathogenicity, can emerge earlier in embryogenesis and affect a wider range of tissues without causing death [35].

FIL is distinct from these two systemic syndromes. The uncontrolled proliferation of abnormal adipocytes in FIL results in the formation of localized fat masses, which also generate secondary soft tissue infiltration and skeletal deformities of varying severity, depending on their rate of progression. Nevertheless, the patient's body parts below the neck are usually uninvolved, and the blood and biochemical tests are within the normal range, so FIL should be considered an isolated and sporadic lesion. In other PIK3CA mutation-associated sporadic overgrowth diseases, p.Glu542Lys, p.Glu545Lys, and p.His1047Arg were responsible for the majority of cases [12]. For example, macrodactyly is caused by the overgrowth of bone and adipose tissues, and more than 90% of cases are caused by these three hotspot mutations [38]; similar proportions have been found in segmental overgrowth of limbs involving muscle and bone [39]. Nevertheless, although all detected mutations in this study have been reported previously, the hotspot mutations accounted for only 39% (7/18), which was far below the commonly considered frequency of hotspot mutations and inconsistent with previous reports on the genetic profile of FIL [4, 5, 40]. This contradicts the previously observed phenomenon that hotspot mutations were more frequent in localized and segmental overgrowth, which may be due to the different pathogenic effects of the PIK3CA variants in the head and trunk. More cases are needed to corroborate this finding, and it would be meaningful to clarify the frequency of each type of mutation in FIL, as this would guide the application of the specific targeted drugs that will be discussed later.

The correlation between specific genotypes and the corresponding phenotypes in FIL remains unclear. Hotspot mutations with greater pathogenicity are thought to be associated with more severe clinical manifestations. As shown by the imaging of patients with the p.His1047Arg mutation, three (75%) of them exhibited extensive adipose infiltration involving the masseter, medial pterygoid muscle, lateral pterygoid muscle, and parotid gland, and the overgrowth and deformity of their maxillofacial skeletons were obvious (Table 2). In addition, imaging of three patients harbouring p.Glu542Lys or p.Glu545Lys showed the involvement of at least two masticatory muscles and the parotid gland with hyperplasia of the zygomatic bone. However, the imaging of patients harbouring nonhotspot missense variants showed that the masticatory muscles and maxillofacial skeletons were affected slightly. We found that hotspot mutations caused more tissues to be affected, including muscle, glands, and bones, but VAF did not appear to be significantly associated with severity. Surprisingly, Patient #11, with more serious clinical characteristics, showed a low VAF of PIK3CA mutations (6.37%), indicating that the conclusion that a higher degree of mosaicism in somatic variant-associated diseases usually leads to a more severe phenotype is debatable [41].

Three patients had the PIK3CA frameshift mutation E110del, and in two of them, identifiable masses were observed at a very young age. We believed that these patients exhibited the rapidly progressive form described by Kang [42], in which extensive hyperplasia can be seen soon after birth, and this suggested that p.Glu110del may have a strong pathogenic capacity. Patient #15, who harboured the p.Glu110del mutation, also had polydactyly and macrodactyly of his feet, which has not been reported before. Only three frameshift mutations (p.Glu109del, p.Glu110del, p.Glu453del) have been detected in PROS, and previously p.Glu110del was mostly seen in CLOVES and KTS with manifestations of vascular malformations [12]. This is the first time that p.Glu110del has been found in FIL, which indicates that the mutation can occur in multiple cell types and is not limited to endothelial cells. However, p.Glu110del occurs in the nonfunctional region of p110α, and the mechanism of its effect on protein function has not been studied as thoroughly as that of other common hotspot mutations, but its specificity still deserves attention.

Molecular genetic diagnosis offers the possibility of targeted therapy for FIL as an alternative to surgery, and the selective PI3K inhibitor alpelisib was approved by the Food and Drug Administration (FDA) in April 2022 for patients with PROS over 2 years of age due to its superior efficacy [43], making it the first potent drug for PROS. Currently, targeted drugs that inhibit molecules of the PI3K-AKT-mTOR pathway have been used in patients with FIL. A 5-year-old girl with FIL taking alpelisib showed sustained improvement over 25 months of treatment, including a reduction in the volume of lipomatosis and hyperpigmentation and a gradual recovery of her oral function, with 3D imaging showing a 14% reduction in facial volume, but the drug was ineffective for skeletal deformities [44]. Another pan-AKT inhibitor, miransertib, was administered in a 5-year-old boy with FIL and HMEG and improved the patient's intractable epilepsy and quality of life [45]. Targeted drugs offer new possibilities for the treatment of FIL: reduction of soft tissue volume and infiltration by oral drugs, followed by surgical adjustment of soft tissues and skeletal deformities. More recently, some mutant-selective drugs have become available that inhibit p110α carrying p.Glu545Lys and p.His1047Arg while minimizing the impact on wild-type p110α, thus avoiding the hyperglycaemia that results from PI3K inhibitors [46]. Characterizing the genetic profile of FIL will provide more data for the design and application of novel mutant-selective targeted drugs, and the availability of such drugs will benefit more patients in the future.

## Conclusions

We identified several rare clinical phenotypes and overgrowth in other body parts of patients with FIL that had not been reported previously. We found four novel PIK3CA variants associated with FIL, including the deletion mutation p.Glu110del, and determined that PIK3CA hotspot mutations lead to more severe infiltration and malformations.

## Methods

### Phenotypic characteristics of the patients

Patients who visited our department from 2014 to 2022 were included in this study, and all patients signed a consent form to use their clinical information for the study. Photographs were taken of all patients, including frontal and lateral facial appearance and photos that exposed the teeth and tongue. Imaging examination is essential for an accurate diagnosis before biopsy and surgery are performed to obtain histological results. CT 3D reconstruction was used to identify potential skeletal deformities and to construct a skeletal model preoperatively. MRI was used to show adipose tissue infiltration as well as soft tissue structures. The primary criteria for diagnosis included (1) congenital facial enlargement and (2) MRI findings of infiltrative growth of facial adipose tissue into surrounding muscles and glands. Secondary criteria incorporated several frequently observed clinical features reported in the literature, including (1) epidermal nevi, capillary blush, macrodontia, and macroglossia [1]; (2) maxillofacial skeletal abnormalities, including skeletal hyperplasia and skeletal malformations, revealed by CT; and (3) enlargement of the occlusal muscles, parotid glands and other soft structures revealed by MRI. Importantly, when only a thickening of the subcutaneous fat layer without infiltrative manifestations was found on imaging, that is, when the muscles and glands on the affected side exhibited the same signal intensity as the contralateral side, the patient was diagnosed with HFH and excluded.

### Acquisition of samples

The samples used for genetic testing were obtained from surgery or biopsy. For patients undergoing surgery (debulking and liposuction), the samples were adipose tissues isolated from the lesion. The type of samples obtained by biopsy was variable; usually, skin with colour changes and a small amount of subcutaneous adipose tissue were selected, and epidermal nevi were particularly appropriate for testing. For patients without recognizable changes in the facial skin, a disposable biopsy punch with a plunger (Integra Miltex, 3 mm) was used to obtain the skin and subcutaneous adipose tissue of the enlarged facial area.

### Next-generation sequencing (NGS)

DNA was extracted from tissue samples using the Qiagen DNA Extraction Kit (Qiagen, #13323). Genomic DNA fragments required splice modifications for sequencing, which was performed by the NEBNext Ultra II DNA Library Prep Kit for Illumina. After completing library establishment, high-throughput sequencing was performed using the Illumina Nova Seq 6000 platform. The customized NGS panel had an average sequencing depth of 10,000× and > 98% coverage. The included disease-associated mutated genes were ACVRL1, GLMN, PIK3CA, AKT1, mTOR, PTEN, MAP2K1, TEK, MAP3K3, GNA11, GNA14, GNAQ, RASA1, EPHB4, SMAD4, STAMBP, KDR, FLT4, KRAS, and BRAF. The DNA sequences of the examined samples were compared with the reference sequence hg19 (GRCh37) and analysed to determine the corresponding mutations.

### Statistical analysis

Statistical analyses were performed using SPSS Statistics V26.0 software. One-way analysis of variance (ANOVA) was used to analyse the genetic data, and *p* < 0.05 was considered statistically significant. Graphs were designed with Prism 8 software and Adobe Illustrator 2020.

## Data Availability

The datasets generated and/or analysed during the current study are not publicly available due (All data used is the patient’s medical history and examination information, which is stored in the hospital and is not publicly available) but are available from the corresponding author on reasonable request.

## Abbreviations

FIL: Facial infiltrating lipomatosis
PI3K: Phosphatidylinositol3-kinase
AKT: Protein kinase B
mTOR: Mammalian target of rapamycin
PIK3CA: Phosphatidylinositol 3-kinase catalytic subunit alpha
PROS: PIK3CA-related overgrowth spectrum
CT: Computed tomography
3D: Three-dimensional
CLOVES: Congenital lipoma overgrowth, vascular malformation, epidermal nevi, scoliosis
MRI: Magnetic resonance imaging
CNS: Central nervous system
VAF: Variant allele fractions
NCBI: National Center for Biotechnology Information
ABD: Adaptor-binding domain
RBD: Ras-binding domain
